# Supplementary material for: Activation function cyclically switchable convolutional neural network model
Source: PeerJ Comput Sci. 2025 Mar 24;11:e2756. doi: 10.7717/peerj-cs.2756 (PMC11948315; doi:10.7717/peerj-cs.2756)
Supplement: Supplemental Information 1 [file peerj-cs-11-2756-s001.docx]

**Highlights of the new method*:**

1. A new model structure called AFCS-CNN has been proposed, which enables cyclical switching of the activation function.
2. Unlike the studies in the literature, instead of an activation function proposal, the ability to switch the activation function with another activation function during neural network training has been adopted.
3. The concept of cyclic activation function switching strategy during model training has been introduced.
4. A first was achieved by designing a model structure that had not been tried before, thanks to instant activation function switches during neural network training.
5. The algorithm of the proposed model structure is designed to allow easy integration of all CNN models into the structure.
6. Training with the proposed model structure has provided superior success in many problems compared to training with fixed activation functions.
7. A state-of-the-art success has been achieved with the proposed model structure in plant seedling classification.
